# Supplementary material for: Structural basis for substrate recognition and chemical inhibition of oncogenic MAGE ubiquitin ligases
Source: Nat Commun. 2020 Oct 1;11:4931. doi: 10.1038/s41467-020-18708-x (PMC7529893; doi:10.1038/s41467-020-18708-x)
Supplement: Supplementary file 1 — Supplementary Information [file 41467_2020_18708_MOESM1_ESM.pdf]

Supplementary information for

Structural basis for substrate recognition and chemical inhibition of oncogenic MAGE ubiquitin ligases

Yang et al.

* Correspondence: Patrick Ryan Potts, Email: Ryan.Potts@stude.org

Supplementary Methods

List of quinoline analogs

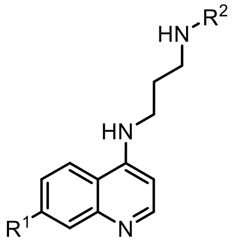

Cpd[a]	SJ Number	R^1	R^2
**5a**	**SJ1008066**	H	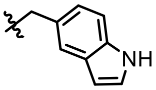
**5b**	**SJ521054**	CF_3	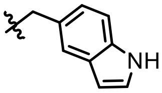
**5c**	**SJ520909**	Cl	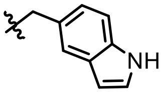
**5d**	**SJ1008065**	Br	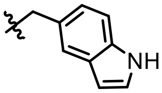
**5e**	**SJ1008067**	CF_3	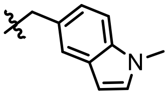
**5f**	**SJ361106**	CF_3	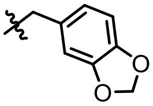
**5g**	**SJ361113**	CF_3	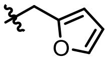
**5h**	**SJ1008068**	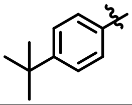	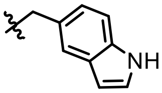
**5i**	**SJ1008069**	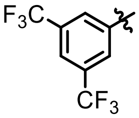	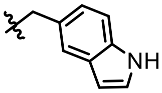
**5j**	**SJ1009807**	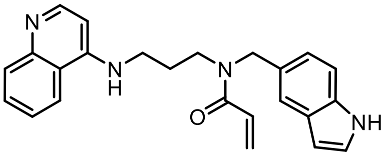	

[a] All compounds prepared as HCl salt form

Experimental Procedure

General. All commercial reagents were used without further purification and the solvents were dried using the Glass Contour Solvent Systems by SG Water USA. All reactions were monitored by UPLC and thin-layer chromatography (TLC) carried out on silica gel coated glass plates. Flash-chromatography was performed on a Biotage Isolera One chromatograpy system using Biotage KP SIL or Biotage KP NH pre-packed columns and the solvent mixture in brackets was used as eluent. Evaporations were carried out *in vacuo* in a Buchi rotary evaporator. Nuclear magnetic resonance (NMR) spectra were obtained on a Bruker Avance II NMR spectrometer at 500 MHz for ^{1}H-NMR spectra and 126 MHz for ^{13}C-NMR spectra. Chemical shifts (ppm) are reported relative to TMS or the solvent peak. Signals are designated as follows: s, singlet; d, doublet; dd, doublet of doublet; t, triplet; q, quadruplet; m, multiplet. Coupling constants (J) are expressed in Hertz.

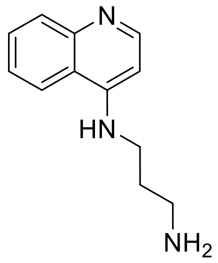

***N*^1^-(quinolin-4-yl)propane-1,3-diamine (3a).** 4-chloroquinoline (0.500 g, 3.06 mmol) and propane-1,3-diamine (1.276 ml, 15.28 mmol) were stirred at 110 °C for 6 h and then cooled at room temperature overnight. Added 1M NaOH (10 mL) and water (5mL) and extracted the aqueous layer with DCM. The DCM layer was washed with water, brine and dried over anhydrous Na_2SO_4, filtered and concentrated to obtain a pale white solid (0.49 g, 80%). The crude product was pure by NMR and used directly for the next step. ^{1}H NMR (500 MHz, Chloroform-*d*) δ 8.54 (d, *J* = 5.3 Hz, 1H), 7.98 – 7.93 (m, 1H), 7.81 – 7.76 (m, 1H), 7.63 – 7.57 (m, 1H), 7.42 – 7.35 (m, 1H), 7.09 (s, 1H), 6.36 (d, *J* = 5.3 Hz, 1H), 3.47 – 3.37 (m, 2H), 3.08 – 2.97 (m, 2H), 1.96 – 1.84 (m, 2H), 1.57 (s, 2H). ^{13}C NMR (126 MHz, $CDCl_3$) δ 151.15, 150.34, 148.46, 129.73, 128.85, 124.32, 120.17, 119.06, 98.12, 43.42, 41.38, 30.39. LR MS (ESI) Exact mass calculated for $C_{12}H_{16}N_3$ $[M + H]^+$ 202.13, found 202.38.

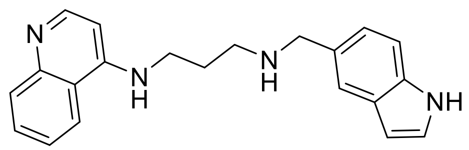

N^1-((1*H*-Indol-5-yl)methyl)-N^3-(quinolin-4-yl)propane-1,3-diamine (5a). N^1-(quinolin-4-yl)propane-1,3-diamine (270 mg, 1.341 mmol) and 1*H*-indole-5-carbaldehyde (195 mg, 1.341 mmol) in MeOH (13 ml) were stirred at 23 °C for 1 h, then sodium borohydride (25.4 mg, 0.671 mmol) was added and continued stirring for 1 h more The reaction mixture was concentrated to remove the solvent, water added and the aqueous layer extracted with ethyl acetate. The ethyl acetate layer was washed with brine, dried over anhydrous Na_2SO_4, filtered and concentrated. The crude product was purified on Biotage NH column using a hexanes-ethyl acetate (0-100%) gradient with MeOH (2%) as additive to provide the product (285 mg, 64%). ^{1}H NMR (500 MHz, Chloroform-*d*) δ 8.51 (t, *J* = 7.5 Hz, 2H), 7.93 (d, *J* = 8.4 Hz, 1H), 7.67 (d, *J* = 8.0 Hz, 2H), 7.63 (s, 1H), 7.55 (t, *J* = 7.6 Hz, 1H), 7.38 (d, *J* = 8.3 Hz, 1H), 7.27 – 7.24 (m, 1H), 7.20 (dd, *J* = 8.3, 1.2 Hz, 1H), 7.14 (t, *J* = 7.4 Hz, 1H), 6.54 (s, 1H), 6.32 (d, *J* = 5.3 Hz, 1H), 3.94 (s, 2H), 3.41 (q, *J* = 5.7 Hz, 2H), 3.05 – 2.96 (m, 2H), 1.95 (p, *J* = 5.8 Hz, 2H), 1.74 (s, 1H). ^{13}C NMR (126 MHz, $CDCl_3$) δ 151.11, 150.58, 148.37, 135.21, 131.13, 129.43, 128.76, 128.07, 124.77, 124.22, 122.91, 120.80, 120.50, 119.16, 111.18, 102.53, 97.95, 54.96, 49.30, 44.10, 27.53. LRMS (ESI) Exact mass calculated for $C_{21}H_{23}N_4$ $[M + H]^+$ 331.19, found 331.51. The product was resuspended in 1.25 M HCl in methanol solution (2 mL) and stirred for 1 h at 23 °C. The solvent and excess HCl were then evaporated *in vacuo* to give the corresponding HCl salt. **(SJ1008066)**.

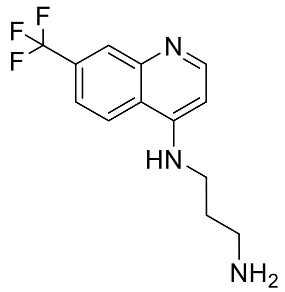

N^1-(7-(Trifluoromethyl)quinolin-4-yl)propane-1,3-diamine (3b). 4-chloro-7-(trifluoromethyl)quinoline (1.3 g, 5.61 mmol) and propane-1,3-diamine (2.343 ml, 28.1 mmol) were stirred at 110 °C for 6 h and then cooled at room temperature overnight. Added 1M NaOH (10 mL) and water (5 mL) and extracted the aqueous layer with DCM. The DCM layer was washed

with water, brine and dried over anhydrous Na_2SO_4, filtered and concentrated to obtain a pale yellow solid. (0.9 g, 60%). The crude product was pure by NMR and used directly for the next step. ^{1}H NMR (500 MHz, Chloroform-*d*) δ 8.58 (d, *J* = 5.3 Hz, 1H), 8.23 (s, 1H), 7.90 (d, *J* = 8.7 Hz, 1H), 7.72 (s, 1H), 7.53 (dd, *J* = 8.7, 1.6 Hz, 1H), 6.40 (d, *J* = 5.4 Hz, 1H), 3.43 (q, *J* = 6.0 Hz, 2H), 3.13 – 3.01 (m, 2H), 1.97 – 1.84 (m, 2H), 1.49 (s, 2H). ^{13}C NMR (126 MHz, $CDCl_3$) δ 152.39, 150.31, 147.74, 131.00, 130.74, 130.48, 130.22, 127.44, 127.41, 127.37, 127.34, 125.21, 123.05, 121.87, 120.88, 119.76, 119.74, 119.71, 119.69, 99.20, 44.00, 41.64, 29.71. LRMS (ESI) $C_{13}H_{15}F_3N_3$ $[M+H]^+$ 270.12, found 270.40.

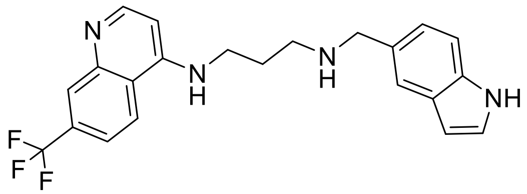

***N*¹-((1H-Indol-5-yl)methyl)-*N*³-(7-(trifluoromethyl)quinolin-4-yl)propane-1,3-diamine (5b).** *N*¹-(7-(trifluoromethyl)quinolin-4-yl)propane-1,3-diamine (135 mg, 0.500 mmol) and 1*H*-indole-5-carbaldehyde (72.6 mg, 0.500 mmol) in MeOH (5 ml) were stirred at 23 °C for 1 h, then sodium borohydride (9.46 mg, 0.250 mmol) was added and continued stirring for 1 h more. The reaction mixture was concentrated to remove the solvent, water added and the aqueous layer extracted with ethyl acetate. The ethyl acetate layer was washed with brine, dried over anhydrous Na_2SO_4, filtered and concentrated. The crude product was purified on Biotage NH column using a DCM-MeOH (0-10%) gradient to give the desired product (83 mg, 42%). ^{1}H NMR (500 MHz, DMSO-*d6*) δ 11.00 (s, 1H), 8.47 (d, *J* = 5.4 Hz, 1H), 8.25 (d, *J* = 8.8 Hz, 1H), 8.05 (s, 1H), 7.85 (s, 1H), 7.49 – 7.45 (m, 2H), 7.34 – 7.28 (m, 2H), 7.09 (dd, *J* = 8.3, 1.5 Hz, 1H), 6.55 (d, *J* = 5.5 Hz, 1H), 6.36 – 6.32 (m, 1H), 3.77 (s, 2H), 3.38 – 3.31 (m, 2H), 2.70 (t, *J* = 6.4 Hz, 2H), 2.31 (s, 1H), 1.85 (p, *J* = 6.5 Hz, 2H). ^{13}C NMR (126 MHz, DMSO-d6) δ 152.32, 149.98, 147.41, 134.98, 131.03, 129.34, 129.09, 128.84, 128.58, 127.56, 127.42, 126.34, 126.30, 126.27, 126.24, 125.30, 125.26, 123.77, 123.09, 121.78, 120.93, 120.85, 119.31, 118.77, 118.75, 118.72, 118.70, 111.00, 100.77, 99.56, 53.81, 46.97, 41.61, 27.59. LRMS (ESI) $C_{22}H_{22}F_3N_4$ $[M+H]^+$ 399.18, found 399.53. The product was resuspended in 1.25 M HCl in methanol solution (2 mL) and stirred for 1 h at 23 °C. The solvent and excess HCl were then evaporated *in vacuo* to give the corresponding HCl salt **(SJ521054)**.

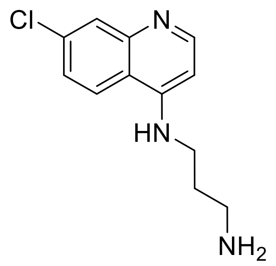

N^1-(7-chloroquinolin-4-yl)propane-1,3-diamine (3c). 4,7-dichloroquinoline (1.000 g, 5.05 mmol) and propane-1,3-diamine (2.107 ml, 25.2 mmol) were stirred at 110 °C for 6 h and then cooled at room temperature overnight. Added 1M NaOH (10 mL) and water (5 mL) and extracted the aqueous layer with DCM. The organic layer was washed with water, brine and dried over anhydrous Na_2SO_4, filtered and concentrated to obtain a pale yellow solid (0.85 g, 71%). The crude product was pure by NMR and used directly for the next step. ^{1}H NMR (500 MHz, Chloroform-*d*) δ 8.51 (d, *J* = 5.4 Hz, 1H), 7.93 (d, *J* = 2.1 Hz, 1H), 7.72 (d, *J* = 8.9 Hz, 1H), 7.45 (s, 1H), 7.32 (dd, *J* = 8.9, 2.2 Hz, 1H), 6.33 (d, *J* = 5.4 Hz, 1H), 3.45 – 3.38 (m, 2H), 3.08 – 3.03 (m, 2H), 1.94 – 1.85 (m, 2H), 1.51 (s, 2H). ^{13}C NMR (126 MHz, $CDCl_3$) δ 152.17, 150.41, 149.21, 134.61, 128.60, 124.94, 121.95, 117.53, 98.29, 43.81, 41.57, 29.92. LRMS (ESI) Exact mass calculated for $C_{12}H_{15}ClN_3$ $[M + H]^+$ 236.10, found 236.27.

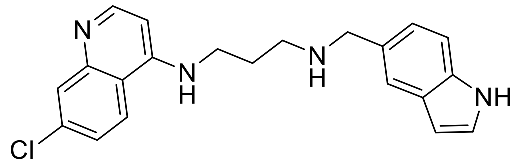

N^1-((1*H*-Indol-5-yl)methyl)-N^3-(7-chloroquinolin-4-yl)propane-1,3-diamine (5c). N^1-(7-chloroquinolin-4-yl)propane-1,3-diamine (200 mg, 0.848 mmol) and 1*H*-indole-5-carbaldehyde (123 mg, 0.848 mmol) in MeOH (8 ml) were stirred at 23 °C for 1 h, then sodium borohydride (16.05 mg, 0.424 mmol) was added and continued stirring for 1 h more The reaction mixture was concentrated to remove the solvent, water added and the aqueous layer extracted with ethyl acetate. The ethyl acetate layer was washed with brine, dried over anhydrous Na_2SO_4, filtered and concentrated. The crude product was purified on Biotage NH column using a hexanes-ethyl acetate (0-100%) gradient with MeOH (2%) as additive to provide the product (158 mg, 51%). ^{1}H NMR (500 MHz, Chloroform-*d*) δ 8.52 (s, 1H), 8.47 (d, *J* = 5.4 Hz, 1H), 7.97 (s, 1H), 7.88 (d, *J* = 2.1 Hz, 1H), 7.61 (s, 1H), 7.48 (d, *J* = 8.9 Hz, 1H), 7.39 (d, *J* = 8.3 Hz, 1H), 7.28 (t, *J* = 2.8 Hz,

1H), 7.18 (dd, J = 8.3, 1.4 Hz, 1H), 6.92 (dd, J = 8.9, 2.2 Hz, 1H), 6.56 – 6.52 (m, 1H), 6.27 (d, J = 5.4 Hz, 1H), 3.92 (s, 2H), 3.42 – 3.36 (m, 2H), 3.06 – 3.00 (m, 2H), 1.98 – 1.91 (m, 2H), 1.74 (s, 1H). ^{13}C NMR (126 MHz, $CDCl_3$) δ 152.03, 150.63, 149.06, 135.23, 134.48, 131.01, 128.20, 128.12, 124.93, 124.85, 122.84, 122.57, 120.56, 117.59, 111.25, 102.53, 98.12, 54.97, 49.56, 44.30, 27.23. LRMS (ESI) Exact mass calculated for $C_{21}H_{22}ClN_4$ $[M + H]^+$ 365.15, found 365.40. The product was resuspended in 1.25 M HCl in methanol solution (2 mL) and stirred for 1 h at 23 °C. The solvent and excess HCl were then evaporated *in vacuo* to give the corresponding HCl salt **(SJ520909)**.

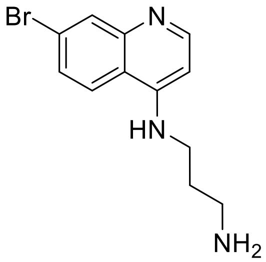

***N*1-(7-Bromoquinolin-4-yl)propane-1,3-diamine (3d).** 7-bromo-4-chloroquinoline (1.000 g, 4.12 mmol) and propane-1,3-diamine (1.721 ml, 20.62 mmol) were stirred at 110 °C for 6 h and then cooled at room temperature overnight. Added 1M NaOH (10 mL) and water (5mL) and extracted the aqueous layer with DCM. The DCM layer was washed with water, brine and dried over anhydrous Na_2SO_4, filtered and concentrated to obtain a pale white solid (0.93 g, 80%). The crude product was pure by NMR and used directly for the next step. ^{1}H NMR (500 MHz, Chloroform-*d*) δ 8.49 (d, J = 5.4 Hz, 1H), 8.11 (d, J = 2.0 Hz, 1H), 7.64 (d, J = 8.9 Hz, 1H), 7.50 – 7.41 (m, 2H), 6.33 (d, J = 5.4 Hz, 1H), 3.41 (q, J = 6.0 Hz, 2H), 3.08 – 3.00 (m, 2H), 1.93 – 1.85 (m, 2H), 1.59 (s, 2H). ^{13}C NMR (126 MHz, $CDCl_3$) δ 152.06, 150.48, 149.42, 131.85, 127.46, 122.86, 122.05, 117.83, 98.36, 43.75, 41.53, 29.90. LRMS (ESI) Exact mass calculated for $C_{12}H_{15}BrN_3$ $[M + H]^+$ 280.04, found 280.20.

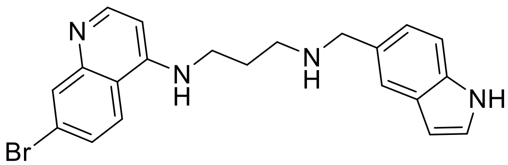

***N*1-((1*H*-Indol-5-yl)methyl)-*N*3-(7-bromoquinolin-4-yl)propane-1,3-diamine (5d).** *N*1-(7-bromoquinolin-4-yl)propane-1,3-diamine (406 mg, 1.449 mmol) and 1*H*-indole-5-carbaldehyde

(210 mg, 1.449 mmol) in MeOH (14 ml) were stirred at 23 °C for 1 h, then sodium borohydride (27.4 mg, 0.725 mmol) was added and continued stirring for 1 h more The reaction mixture was concentrated to remove the solvent, water added and the aqueous layer extracted with ethyl acetate. The ethyl acetate layer was washed with brine, dried over anhydrous Na_2SO_4, filtered and concentrated. The crude product was purified on Biotage NH column using a hexanes-ethyl acetate (0-100%) gradient with MeOH (2%) as additive to provide the product (452 mg, 76%). ^{1}H NMR (500 MHz, Chloroform-*d*) δ 8.47 (d, *J* = 5.3 Hz, 1H), 8.35 (s, 1H), 8.06 (d, *J* = 2.0 Hz, 1H), 7.98 (s, 1H), 7.61 (s, 1H), 7.40 (dd, *J* = 8.6, 4.3 Hz, 2H), 7.29 (t, *J* = 2.8 Hz, 1H), 7.18 (d, *J* = 8.3 Hz, 1H), 7.05 (dd, *J* = 8.9, 2.0 Hz, 1H), 6.56 – 6.53 (m, 1H), 6.29 (d, *J* = 5.4 Hz, 1H), 3.92 (s, 2H), 3.40 (q, *J* = 5.5 Hz, 2H), 3.08 – 2.99 (m, 2H), 1.99 – 1.92 (m, 2H), 1.64 (s, 1H). ^{13}C NMR (126 MHz, $CDCl_3$) δ 152.03, 150.66, 149.39, 135.20, 131.58, 131.06, 128.12, 127.38, 124.90, 122.86, 122.75, 122.61, 120.59, 117.91, 111.23, 102.59, 98.20, 54.98, 49.59, 44.33, 27.25. LRMS (ESI) Exact mass calculated for $C_{21}H_{22}BrN_4$ $[M + H]^+$ 409.10, found 409.33. The product was resuspended in 1.25 M HCl in methanol solution (2 mL) and stirred for 1 h at 23 °C. The solvent and excess HCl were then evaporated *in vacuo* to give the corresponding HCl salt **(SJ1008065)**.

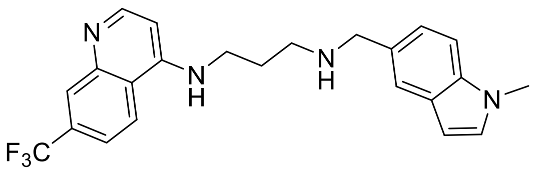

***N*^{1}-((1-Methyl-1*H*-indol-5-yl)methyl)-*N*^{3}-(7-(trifluoromethyl)quinolin-4-yl)propane-1,3-diamine (5e).** *N*^{1}-(7-(trifluoromethyl)quinolin-4-yl)propane-1,3-diamine (100 mg, 0.371 mmol) and 1-methyl-1H-indole-5-carbaldehyde (59.1 mg, 0.371 mmol) in MeOH (3.5 ml) were stirred at 23 °C for 1 h, then sodium borohydride (7.02 mg, 0.186 mmol) was added and continued stirring for 1 h more The reaction mixture was concentrated to remove the solvent, water added and the aqueous layer extracted with ethyl acetate. The ethyl acetate layer was washed with brine, dried over anhydrous Na_2SO_4, filtered and concentrated. The crude product was purified on Biotage NH column using a hexanes-ethyl acetate (0-100%) gradient to provide the product (87 mg, 57%). ^{1}H NMR (500 MHz, Chloroform-*d*) δ 8.53 (d, *J* = 5.3 Hz, 1H), 8.22 (s, 1H), 8.16 (s, 1H), 7.60 (s, 1H), 7.56 (d, *J* = 8.7 Hz, 1H), 7.31 (d, *J* = 8.3 Hz, 1H), 7.20 (dd, *J* = 8.3, 1.5 Hz, 1H), 7.12 (d, *J* = 3.1 Hz, 1H), 7.03 (dd, *J* = 8.7, 1.7 Hz, 1H), 6.46 (d, *J* = 3.0 Hz, 1H), 6.34 (d, *J* = 5.4 Hz, 1H), 3.93 (s, 2H), 3.83 (s, 3H), 3.41 (q, *J* = 5.5 Hz, 2H), 3.09 – 3.04 (m, 2H), 2.00 – 1.93 (m, 2H), 1.67 (s,

1H). ^{13}C NMR (126 MHz, $CDCl_3$) δ 152.31, 150.44, 147.61, 136.14, 130.77, 130.50, 130.25, 130.00, 129.56, 128.70, 127.07, 127.04, 127.00, 126.97, 125.24, 123.08, 122.41, 122.40, 120.94, 120.75, 119.70, 119.68, 119.65, 119.62, 109.46, 100.84, 98.95, 54.96, 49.71, 44.44, 32.89, 27.15. LRMS (ESI) $C_{23}H_{24}F_3N_4$ $[M+H]^+$ 413.20, found 413.44. The product was resuspended in 1.25 M HCl in methanol solution (2 mL) and stirred for 1 h at 23 °C. The solvent and excess HCl were then evaporated *in vacuo* to give the corresponding HCl salt **(SJ1008067)**.

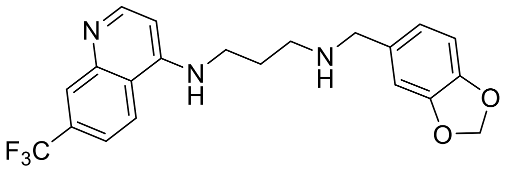

***N*^{1}-(Benzo[*d*][1,3]dioxol-5-ylmethyl)-*N*^{3}-(7-(trifluoromethyl)quinolin-4-yl)propane-1,3-diamine (5f).** *N*^{1}-(7-(trifluoromethyl)quinolin-4-yl)propane-1,3-diamine (100 mg, 0.371 mmol) and benzo[*d*][1,3]dioxole-5-carbaldehyde (55.8 mg, 0.371 mmol) in MeOH (3.5 ml) were stirred at 23 °C for 1 h, then sodium borohydride (7.02 mg, 0.186 mmol) was added and continued stirring for 1 h more. The reaction mixture was concentrated to remove the solvent, water added and the aqueous layer extracted with ethyl acetate. The ethyl acetate layer was washed with brine, dried over anhydrous Na_2SO_4, filtered and concentrated. The crude product was purified on Biotage NH column using a hexanes-ethyl acetate (0-100%) gradient to obtain the product (63.5 mg, 42%). ^{1}H NMR (500 MHz, Chloroform-*d*) δ 8.58 (d, J = 5.3 Hz, 1H), 8.22 (s, 1H), 7.83 (s, 1H), 7.73 (d, J = 8.7 Hz, 1H), 7.42 (d, J = 8.7 Hz, 1H), 6.85 (s, 1H), 6.82 – 6.77 (m, 2H), 6.38 (d, J = 5.3 Hz, 1H), 5.99 (s, 2H), 3.75 (s, 2H), 3.42 (q, J = 5.0 Hz, 2H), 3.03 – 2.96 (m, 2H), 1.99 – 1.92 (m, 2H), 1.68 (s, 1H). ^{13}C NMR (126 MHz, $CDCl_3$) δ 152.40, 150.29, 147.92, 147.71, 146.95, 133.45, 130.98, 130.72, 130.46, 130.20, 127.39, 127.37, 127.34, 127.31, 125.23, 123.06, 122.04, 121.58, 120.89, 119.75, 119.73, 119.70, 119.68, 109.02, 108.32, 101.13, 99.20, 54.32, 49.43, 44.24, 27.32. LRMS (ESI) $C_{21}H_{21}F_3N_3O_2$ $[M+H]^+$ 404.16, found 404.39. The product was resuspended in 1.25 M HCl in methanol solution (2 mL) and stirred for 1 h at 23 °C. The solvent and excess HCl were then evaporated *in vacuo* to give the corresponding HCl salt **(SJ361106)**.

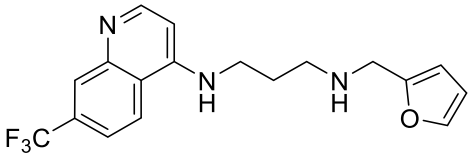

***N*[1]-(Furan-2-ylmethyl)-*N*[3]-(7-(trifluoromethyl)quinolin-4-yl)propane-1,3-diamine (5g).** N^1-(7-(trifluoromethyl)quinolin-4-yl)propane-1,3-diamine (100 mg, 0.371 mmol) and furan-2-carbaldehyde (35.7 mg, 0.371 mmol) in MeOH (3.5 ml) were stirred at 23 °C for 1 h then sodium borohydride (7.02 mg, 0.186 mmol) was added and continued stirring for 1 h more. The reaction mixture was concentrated to remove the solvent, water added and the aqueous layer extracted with ethyl acetate. The ethyl acetate layer was washed with brine, dried over anhydrous Na_2SO_4, filtered and concentrated. The crude product was purified on Biotage NH column using a hexanes-ethyl acetate (0-100%) gradient to obtain the product (107 mg, 82%). ^{1}H NMR (500 MHz, Chloroform-*d*) δ 8.58 (d, *J* = 5.3 Hz, 1H), 8.22 (s, 1H), 7.91 (s, 1H), 7.82 (d, *J* = 8.7 Hz, 1H), 7.44 (dd, *J* = 8.7, 1.5 Hz, 1H), 7.41 – 7.38 (m, 1H), 6.40 – 6.35 (m, 2H), 6.24 (d, *J* = 3.1 Hz, 1H), 3.87 (s, 2H), 3.42 (q, *J* = 5.7 Hz, 2H), 3.01 – 2.96 (m, 2H), 1.99 – 1.92 (m, 2H), 1.70 (s, 1H). ^{13}C NMR (126 MHz, $CDCl_3$) δ 153.08, 152.40, 150.37, 147.74, 142.21, 130.96, 130.70, 130.44, 130.18, 127.40, 127.37, 127.33, 127.30, 125.24, 123.07, 122.14, 120.93, 119.68, 119.65, 119.63, 119.60, 110.33, 107.37, 99.15, 49.05, 46.23, 44.28, 27.02. LRMS (ESI) $C_{18}H_{19}F_3N_3O$ $[M+H]^+$ 350.15, found 350.43. The product was resuspended in 1.25 M HCl in methanol solution (2 mL) and stirred for 1 h at 23 °C. The solvent and excess HCl were then evaporated *in vacuo* to give the corresponding HCl salt **(SJ361113)**.

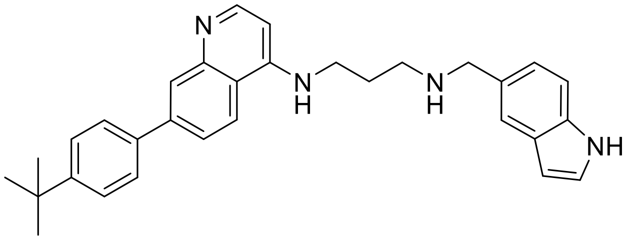

***N*[1]-((1H-Indol-5-yl)methyl)-*N*[3]-(7-(4-(tert-butyl)phenyl)quinolin-4-yl)propane-1,3-diamine (5h).** N^1-((1*H*-indol-5-yl)methyl)-N^3-(7-bromoquinolin-4-yl)propane-1,3-diamine (55 mg, 0.134 mmol), (4-(tert-butyl)phenyl)boronic acid (47.8 mg, 0.269 mmol) and potassium phosphate (62.7 mg, 0.296 mmol) in dioxane (1.5 ml) and water (0.250 ml) were degassed under N_2 and then Pd(dppf)Cl_2 (19.66 mg, 0.027 mmol) was added. The reaction mixture was heated at 110 °C under N_2 for 16 h and then cooled to room temperature. Water was added, extracted with DCM and the DCM layer was washed with brine, dried over anhydrous Na_2SO_4, filtered and concentrated to obtain a dark brown product. The crude product was purified on a Biotage 11g NH column using

a DCM-MOH (0-8% MeOH) gradient to provide the desired product (30 mg, 48%). ^{1}H NMR (500 MHz, Chloroform-*d*) δ 8.52 (d, *J* = 5.3 Hz, 1H), 8.36 (s, 1H), 8.16 (s, 1H), 7.71 (s, 1H), 7.68 – 7.61 (m, 4H), 7.51 (d, *J* = 8.3 Hz, 2H), 7.40 (d, *J* = 8.3 Hz, 1H), 7.34 (dd, *J* = 8.6, 1.6 Hz, 1H), 7.27 (t, *J* = 2.7 Hz, 1H), 7.22 (d, *J* = 8.3 Hz, 1H), 6.56 (s, 1H), 6.31 (d, *J* = 5.3 Hz, 1H), 3.96 (s, 2H), 3.43 (q, *J* = 5.3 Hz, 2H), 3.10 – 2.97 (m, 2H), 2.02 – 1.90 (m, 2H), 1.65 (s, 1H), 1.39 (s, 9H). ^{13}C NMR (126 MHz, $CDCl_3$) δ 151.53, 150.62, 150.50, 148.79, 141.01, 137.57, 135.22, 131.26, 128.12, 126.91, 126.82, 125.82, 124.74, 123.52, 122.94, 121.31, 120.58, 118.13, 111.22, 102.68, 97.90, 54.95, 49.37, 44.14, 34.61, 31.40, 27.57. LRMS (ESI) $C_{31}H_{35}N_4$ $[M+H]^+$ 463.29, found 463.45. The product was resuspended in 1.25 M HCl in methanol solution (2 mL) and stirred for 1 h at 23 °C. The solvent and excess HCl were then evaporated *in vacuo* to give the corresponding HCl salt **(SJ1008068)**.

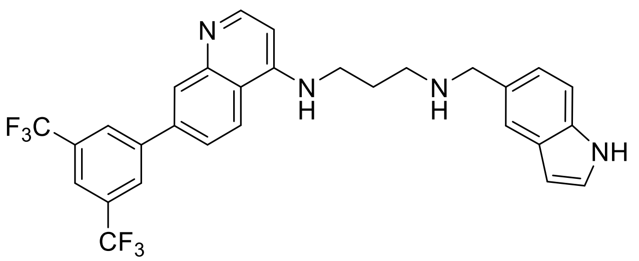

***N*¹-((1*H*-Indol-5-yl)methyl)-*N*³-(7-(3,5-bis(trifluoromethyl)phenyl)quinolin-4-yl)propane-1,3-diamine (5i).** *N*¹-((1*H*-indol-5-yl)methyl)-*N*³-(7-bromoquinolin-4-yl)propane-1,3-diamine (55 mg, 0.134 mmol), (3,5-bis(trifluoromethyl)phenyl)boronic acid (69.3 mg, 0.269 mmol) and potassium phosphate (62.7 mg, 0.296 mmol) in dioxane (1.5 ml) and water (0.250 ml) were degassed under N_2 and then $Pd(dppf)Cl_2$ (19.66 mg, 0.027 mmol) was added and the reaction mixture was heated at 110 °C under N_2 for 16 h and then cooled to room temperature. Water was added, extracted with DCM and the DCM layer was washed with brine, dried over anhydrous Na_2SO_4, filtered and concentrated to obtain a dark brown product. The crude product was purified on a Biotage 11g NH column using a DCM-MOH (0-8% MeOH) gradient to provide the desired product (32 mg, 44%). ^{1}H NMR (500 MHz, Chloroform-*d*) δ 8.53 (d, *J* = 5.3 Hz, 1H), 8.29 (s, 2H), 8.15 (d, *J* = 1.7 Hz, 1H), 8.09 (s, 2H), 7.88 (s, 1H), 7.70 (s, 1H), 7.54 (d, *J* = 8.6 Hz, 1H), 7.42 (d, *J* = 8.3 Hz, 1H), 7.29 (t, *J* = 2.7 Hz, 1H), 7.20 (d, *J* = 8.3 Hz, 1H), 6.96 (dd, *J* = 8.6, 1.8 Hz, 1H), 6.56 (s, 1H), 6.32 (d, *J* = 5.4 Hz, 1H), 3.96 (s, 2H), 3.46 (t, *J* = 5.6 Hz, 2H), 3.16 – 3.08 (m, 2H), 2.04 – 1.96 (m, 2H), 1.84 (s, 1H). ^{13}C NMR (126 MHz, $CDCl_3$) δ 151.97, 150.58, 148.48,

142.57, 137.76, 135.24, 132.48, 132.21, 131.95, 131.69, 131.22, 128.24, 127.97, 127.51, 127.26, 127.24, 126.73, 124.98, 124.72, 124.57, 122.85, 122.61, 122.52, 122.40, 122.01, 121.12, 121.09, 121.06, 121.03, 121.00, 120.69, 120.23, 119.72, 119.20, 111.30, 102.68, 98.26, 54.90, 50.00, 44.50, 27.19. LRMS (ESI) $C_{29}H_{25}F_6N_4$ $[M+H]^+$ 543.20, found 543.55. The product was resuspended in 1.25 M HCl in methanol solution (2 mL) and stirred for 1 h at 23 °C. The solvent and excess HCl were then evaporated *in vacuo* to give the corresponding HCl salt **(SJ1008069)**.

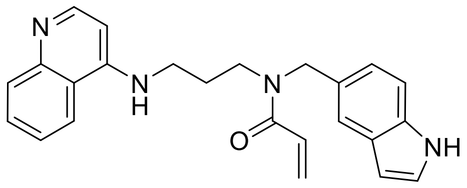

***N*-((1*H*-Indol-5-yl)methyl)-*N*-(3-(quinolin-4-ylamino)propyl)acrylamide (5j).** To *N*[1]-((1*H*-indol-5-yl)methyl)-*N*[3]-(quinolin-4-yl)propane-1,3-diamine (50 mg, 0.151 mmol) in DCM (1.5 ml) was added triethylamine (0.042 ml, 0.303 mmol). The reaction mixture was cooled to 0 °C and acryloyl chloride (0.012 ml, 0.151 mmol) was added and stirred under N_2 for 2 h at 0 °C. Added water to the reaction mixture and extracted the aqueous layer with DCM. The DCM layer was washed with brine, dried over anhydrous Na_2SO_4, filtered and concentrated. The crude product was purified on a Biotage 11g NH column using a DCM-MeOH (0-8% MeOH) gradient to obtain the desired product (6.2 mg, 11%). ^{1}H NMR (500 MHz, Chloroform-*d*) δ 8.49 (d, *J* = 5.3 Hz, 2H), 8.04 (d, *J* = 8.2 Hz, 1H), 7.94 (d, *J* = 8.4 Hz, 1H), 7.64 – 7.58 (m, 1H), 7.49 – 7.41 (m, 2H), 7.36 (d, *J* = 8.4 Hz, 1H), 7.26 – 7.24 (m, 1H), 7.00 (dd, *J* = 8.4, 1.4 Hz, 1H), 6.72 (dd, *J* = 16.7, 10.4 Hz, 1H), 6.61 (t, *J* = 5.7 Hz, 1H), 6.52 (d, *J* = 2.0 Hz, 1H), 6.35 (d, *J* = 5.4 Hz, 1H), 5.77 (dd, *J* = 10.4, 1.9 Hz, 1H), 4.72 (s, 2H), 3.63 (t, *J* = 6.2 Hz, 2H), 3.35 (q, *J* = 6.0 Hz, 2H), 1.90 – 1.81 (m, 2H), 1.73 (s, 1H). ^{13}C NMR (126 MHz, $CDCl_3$) δ 167.97, 150.93, 149.93, 148.63, 135.39, 129.55, 129.07, 128.99, 128.19, 127.88, 127.46, 125.22, 124.61, 120.59, 120.38, 119.26, 118.66, 111.69, 102.56, 98.05, 51.76, 43.22, 39.29, 25.95. LRMS (ESI) $C_{24}H_{25}N_4O$ $[M+H]^+$ 385.20, found 385.47. The product was resuspended in 1.25 M HCl in methanol solution (2 mL) and stirred for 1 h at 23 °C. The solvent and excess HCl were then evaporated *in vacuo* to give the corresponding HCl salt **(SJ1009807)**.

Supplementary Figure 1

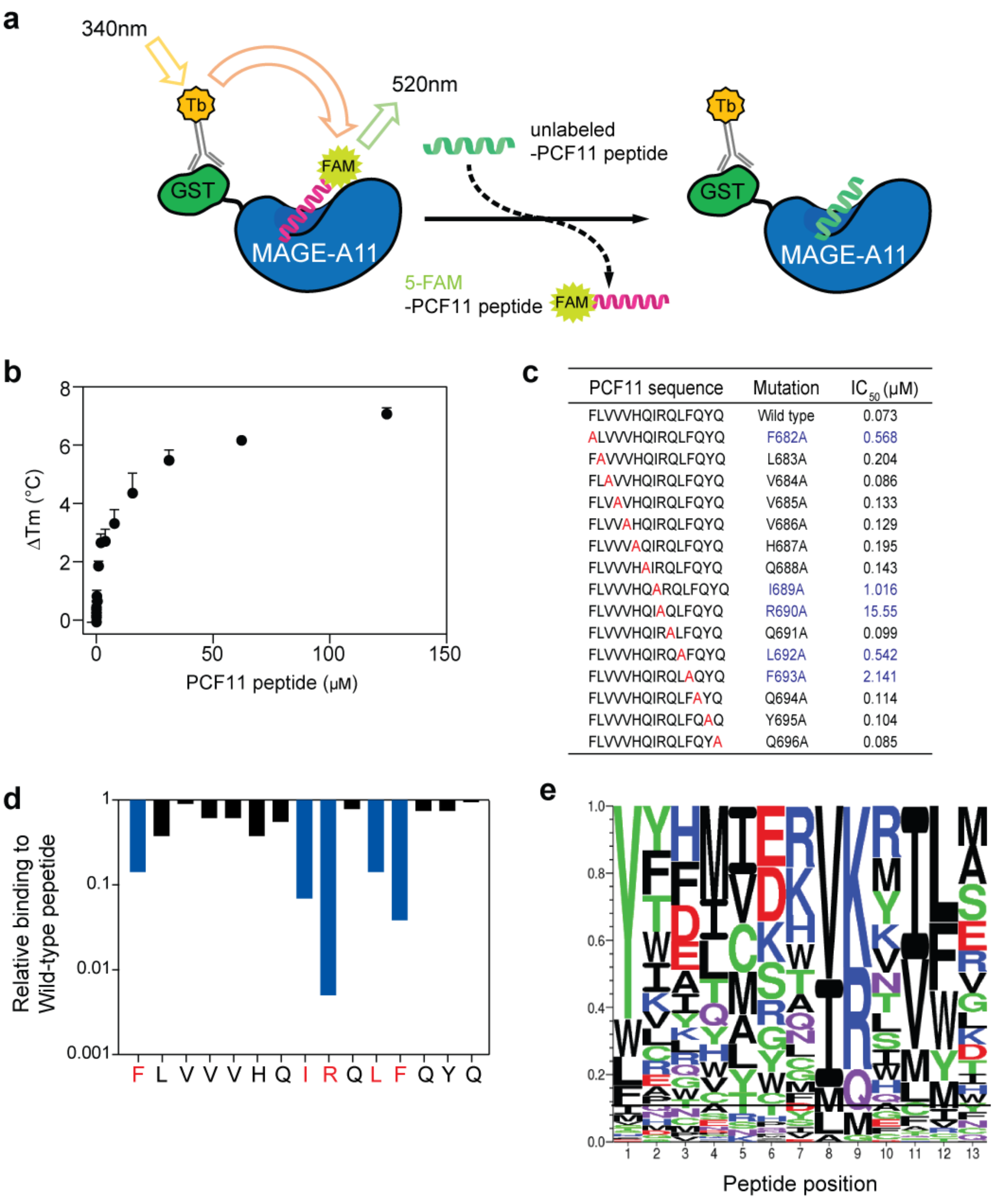

PCF11 sequence	Mutation	IC_{50} (μM)
FLVVVHQIRQLFQYQ	Wild type	0.073
ALVVVHQIRQLFQYQ	F682A	0.568
FAVVVHQIRQLFQYQ	L683A	0.204
FLAVVHQIRQLFQYQ	V684A	0.086
FLVAVHQIRQLFQYQ	V685A	0.133
FLVVAHQIRQLFQYQ	V686A	0.129
FLVVVAQIRQLFQYQ	H687A	0.195
FLVVVHAIRQLFQYQ	Q688A	0.143
FLVVVHQARQLFQYQ	I689A	1.016
FLVVVHQIAQLFQYQ	R690A	15.55
FLVVVHQIRALFQYQ	Q691A	0.099
FLVVVHQIRQAFQYQ	L692A	0.542
FLVVVHQIRQLAQYQ	F693A	2.141
FLVVVHQIRQLFAYQ	Q694A	0.114
FLVVVHQIRQLFQAQ	Y695A	0.104
FLVVVHQIRQLFQYA	Q696A	0.085

Supplementary Figure 1: Identification of the degron motif in PCF11 required for MAGE-A11 binding.

a, Schematic of the TR-FRET MAGE-A11:PCF11 peptide binding assay. To monitor the MAGE-A11 and PCF11 interaction, recombinant GST-tagged MAGE-A11 MHD was incubated with FAM-labeled and unlabeled series of PCF11 peptides and TR-FRET signal was quantified. **b,** Tm-shift in Fig. 1d was analyzed by plotting the temperature at 50% relative fluorescence (n=3 biologically independent experiments per concentration). Data are mean ± SD. **c-d,** Schematic representation of alanine scanning mutations in PCF11 degron peptide. The amino acid sequences of PCF11 degron are listed with alanine substitutions for each residue. IC_{50} values for each mutant by TR-FRET **(c)** and relative binding **(d)** are shown. **e,** MAGE-A11 binding motif sequence and weighted position matrix is shown.

Supplementary Figure 2

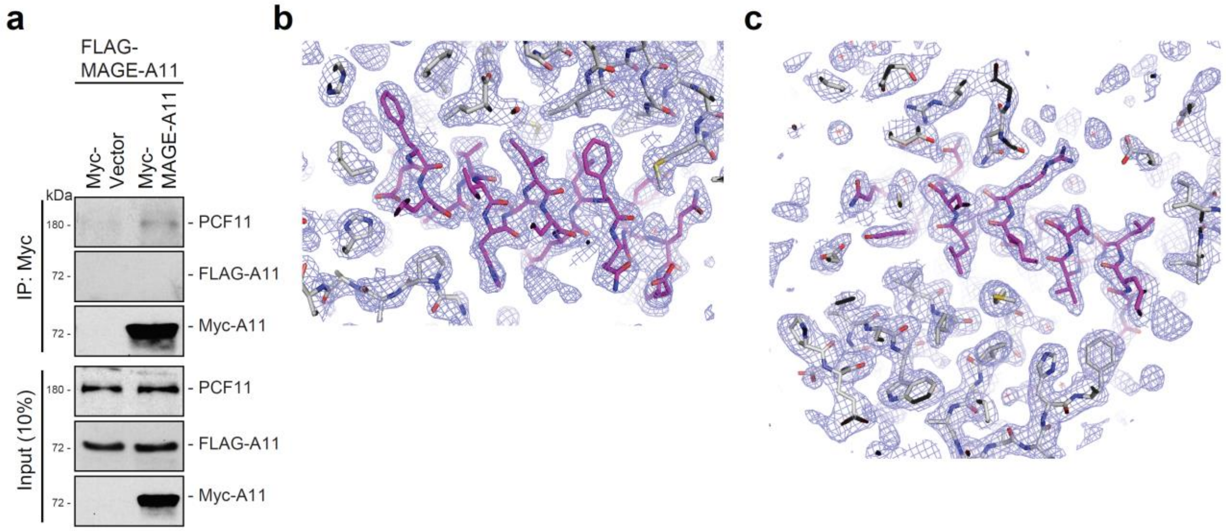

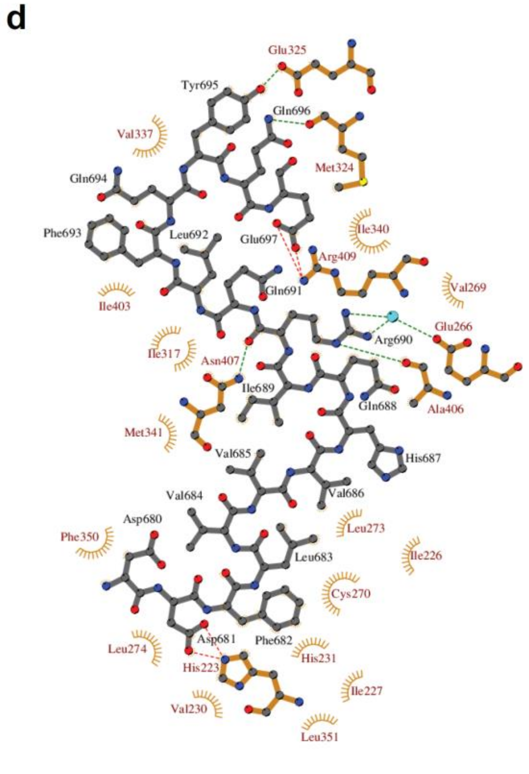

e

Peptide	IC_{50} (nM)
PCF11 WT (FLVVVHQIRQLFQ)	60.4
Phenylglycine	327.3
Homo Phenylalanine	5
2-Fluoro-Phenylalanine	2.6
3-Fluoro-Phenylalanine	8.2
4-Fluoro-Phenylalanine	15.4
2-Chloro-Phenylalanine	3.8
3-Chloro-Phenylalanine	10.1
4-Chloro-Phenylalanine	9.9
2-Trifluoromethyl-Phenylalanine	10.5
3-Trifluoromethyl-Phenylalanine	2.5
4-Trifluoromethyl-Phenylalanine	77.3
2-Cyano-Phenylalanine	2.6
3-Cyano-Phenylalanine	54.4
4-Cyano-Phenylalanine	662.8
2-Methyl-Phenylalanine	12.8
3-Methyl-Phenylalanine	1.7
4-Methyl-Phenylalanine	41.5

Supplementary Figure 2: Characterization of MAGE-A11 SBC for recognition of PCF11. **a,** MAGE-A11 does not form dimers in cell lysates. HEK293FT cells stably expressing FLAG-MAGE-A11 were transfected with Myc-vector or Myc-MAGE-A11 for 48hr and then subjected to IP with anti-Myc followed by SDS-PAGE and immunoblotting for anti-FLAG and anti-PCF11. Source data are provided as a Source Data file. **b,c,** Representative electron-density maps of PCF11 peptide bound to MAGE-A11. Shown are simulated annealing $2F_o - F_c$ composite omit maps

contoured at 1σ. **d,** 2D representation of MAGE-A11-PCF11 interactions by Ligplot[+1]. **e,** TR-FRET binding assay IC_{50} results of GST-MAGE-A11 with PCF11 peptide (FLVVVHQIRQLF*Q) containing the indicated phenylalanine substitutions at the indicated position.

Supplementary Figure 3

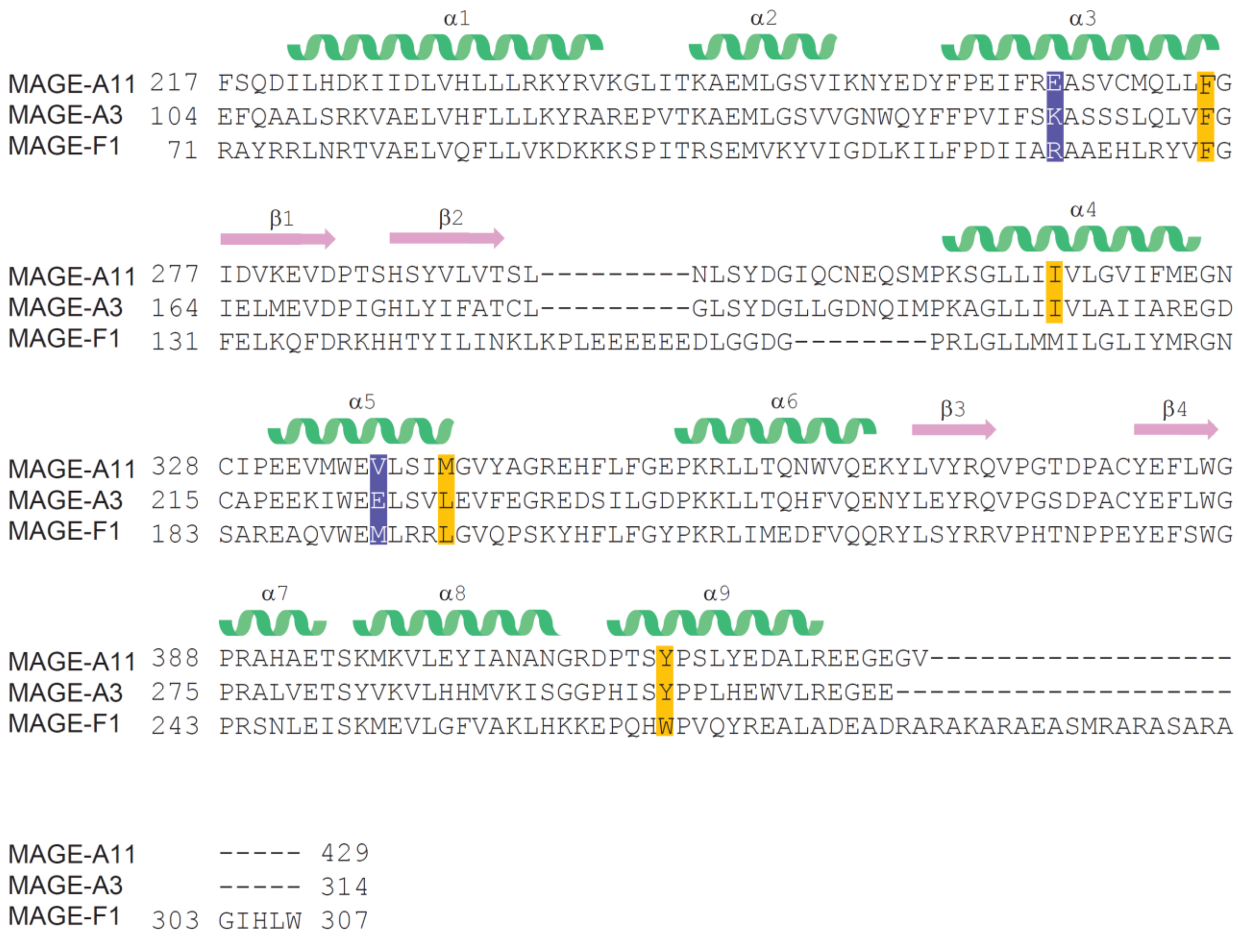

Supplementary Figure 3: Sequence alignment of MAGE-A3, -A11 and -F1 MHDs.

Sequence alignment and secondary structure of MHDs from MAGE-A11, -A3 and -F1. Key conserved interaction residues within the SBC chosen for mutagenesis are shown. Residues in blue highlight substitutions within the SBC that likely contribute to substrate binding selectivity.

Supplementary Figure 4

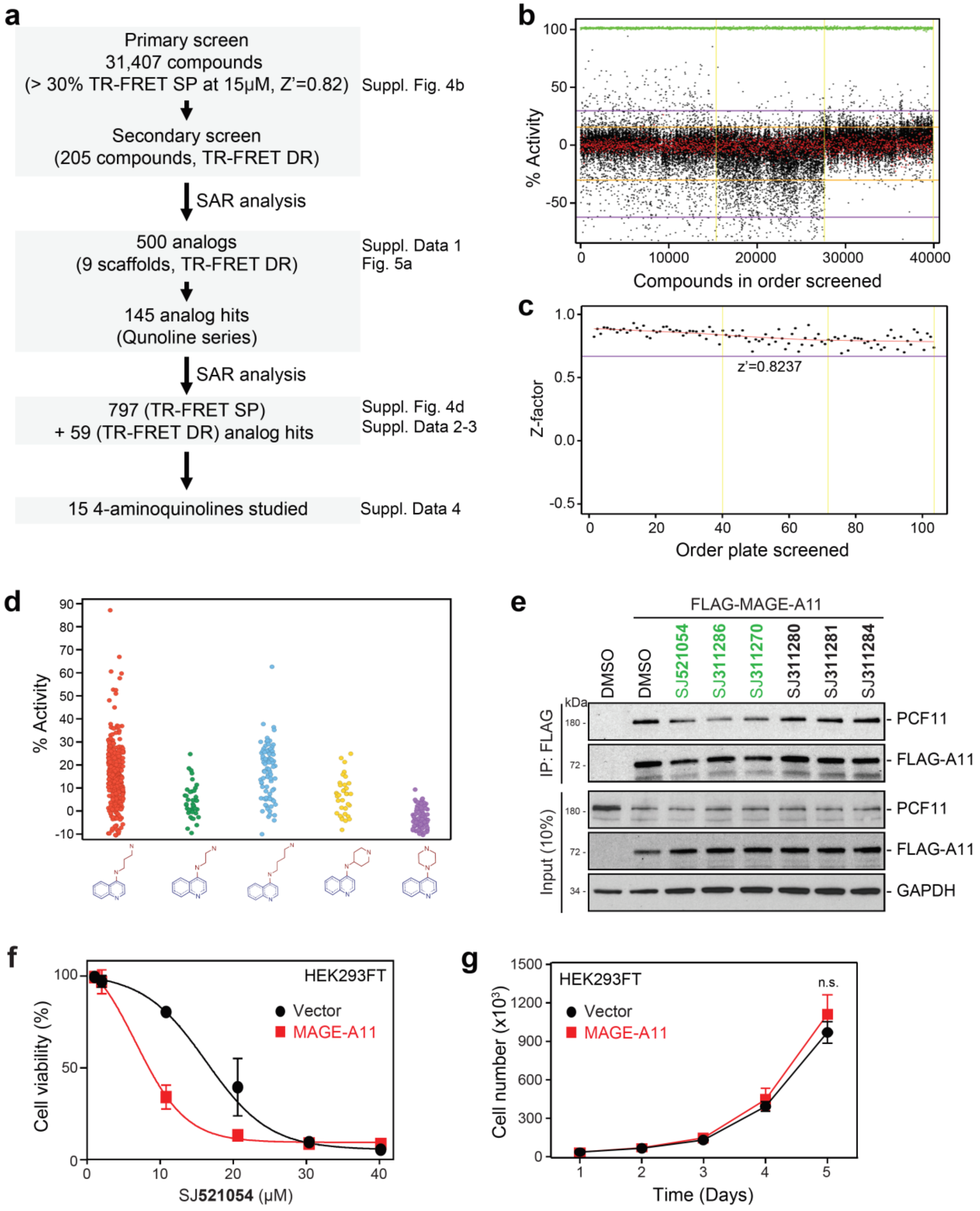

Supplementary Figure 4: Chemical screen for small molecular inhibitors of MAGE-A11:PCF11 interaction.

a. Summary of high-throughput screening approach. **b,** Results of primary screen for all 31,407 compounds screened at one concentration (15 μM) by TR-FRET assay. Positive controls (green); negative control (DMSO, red), screening compounds (black); 5-95% (orange line) and 1-99% (purple line) activity percentiles of the screening compound population are shown. **c,** Z'-factor in the order of plates screened in the primary screen is shown. **d,** Percentage activity scatterplot for 797 analogs from five quinoline series screened at single point (15 μM) by TR-FRET assay. **e,** FLAG-MAGE-A11 stably expressing HEK293FT cell lysates were incubated with 100 μM of the indicated quinoline compounds for 24 hours and then subjected to pull-down with anti-FLAG followed by SDS-PAGE and immunoblotting for endogenous PCF11. Note **SJ521054** (0.40 μM), **SJ311286** (1.16 μM) and **SJ311270** (0.58 μM) are active inhibitors by TR-FRET and **SJ311280** (4.07 μM), **SJ311281** (5.58 μM) and **SJ311284** (20.60 μM) show low activity by TR-FRET. Source data are provided as a Source Data file. **f,** Treatment of **SJ521054** reduces viability of MAGE-A11 expressing HEK293FT cells. HEK293FT cells stably expressing FLAG-vector (black circle) or FLAG-MAGE-A11 (red square) were treated with the indicated concentrations of **SJ521054** and cell viability was measured by alamarBlue assay 24 hours later (n=3 biologically independent experiments per group). Data are mean ± SD. **g,** Overexpression of MAGE-A11 does not affect proliferation of HEK293FT cells. HEK293FT cells stably expressing FLAG-vector (black circle) or FLAG-MAGE-A11 (red square) were counted for cell proliferation at the indicated time points (n=3 biologically independent experiments per group). Data are mean ± SEM. *p*-value by two-way ANOVA followed by Sidak's multiple comparisons test is indicated.

Supplementary Figure 5

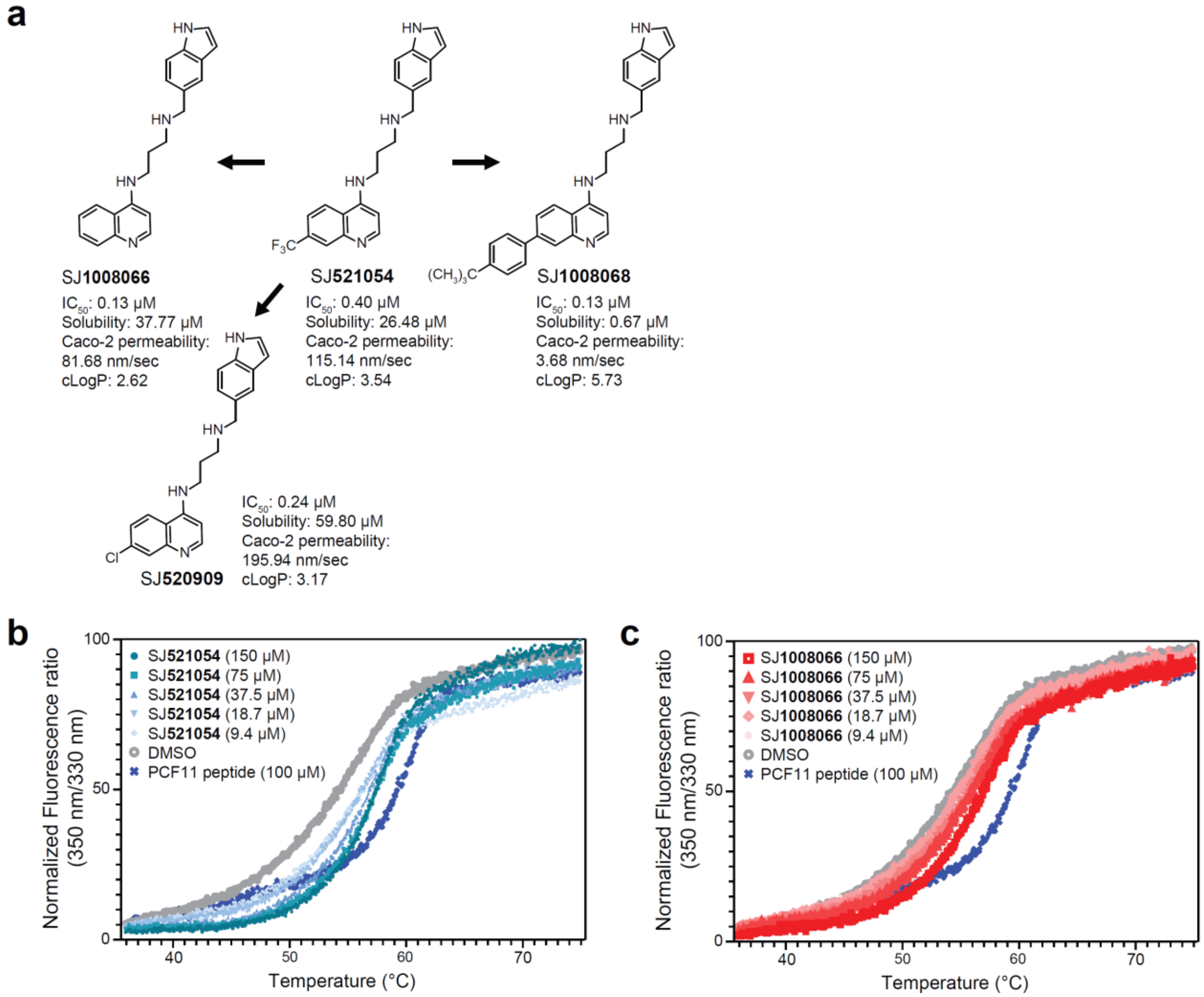

Supplementary Figure 5: SJ521054 and SJ1008066 directly bind to MAGE-A11 MHD.

a, Chemical structure of the **SJ521054** derivative compounds. IC_{50}, solubility, Caco-2 permeability and cLogP values of each compound are shown. **b,c,** Binding of **SJ521054** and **SJ1008066** were evaluated by NanoDSF using the intrinsic fluorescence of His-SUMO-MAGE-A11 MHD. Normalized by fluorescence ratios at 350 nm and 330 nm are shown.

Supplementary Figure 6

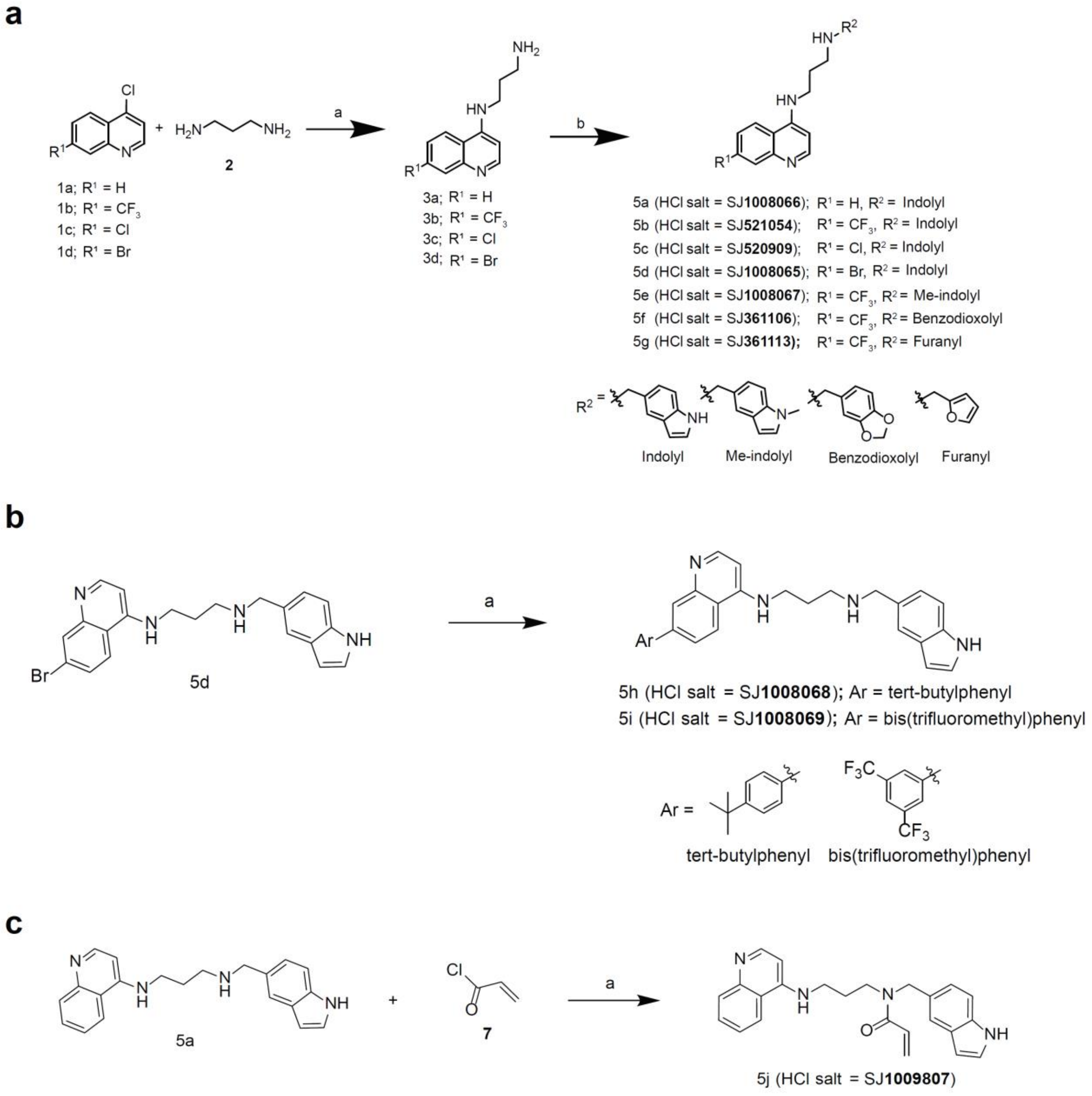

Supplementary Figure 6: Chemical synthesis of compounds in Fig. 5

A typical synthetic routes used to explore the structure–activity relationship of quinoline analogs are shown in **a-c**. Chloroquinoline 1a-1d were condensed with 1,3-propyldiamine (2) to afford the diamine compounds 3a-3d in **a** ([a] Reagents and conditions: (a) 110 °C, 6 h; (b) R^2CHO (**4**), MeOH, 23 °C, 1h, then $NaBH_4$, 23 °C, 1h.)[40]. Reaction of compounds 3a-3d with aldehydes 4 followed by reduction with sodium borohdride provided the quinoline analogs 5a-5g[41]. Suzuki coupling of compound 5d with aryl boronic acids 6 generated substituted quionlines 5h and 5i in **b** ([a] Reagents and conditions: (a) $ArB(OH)_2$ (**6**), $Pd(dppf)Cl_2$, K_3PO_4, dioxane, H_2O (6:1), 110 °C, 16 h.).

Acylation of compound 5a with acryloyl chloride (7) provided the substituted quinoline 5j with a covalent warhead in **c** ([a] Reagents and conditions: (a) TEA, DCM, 0 °C, 2 h.).[42] Analogs 5a-5j were converted to the HCl salts by treatment with methanolic solution of hydrochloric acid. Detail protocols for the synthesis of quinoline analogs are described in full in the supplementary methods.

Supplementary Table 1: Polar and hydrophobic PCF11 interactions with MAGE-A11.

PCF11 Residue*	MAGE-A11 Residue (Hydrophobic Contacts)	MAGE-A11 Residue (Polar, Hydrogen Bond)
D681		H223 NE2
F682	F275, H231, C270, F350, L351, V230	
L683	I226, I227, C270	
V684		
V685	L274, F350	
V686	L273, L274, F275, C270	
H687		
Q688		
I689	L273, L274, I317, M341	
R690	L269, L273	E266 (water-mediated), A406 backbone carbonyl-R690 NE, N407 – R690 backbone carbonyl
Q691		
L692	I317, V337, L338, I340, M341	
F693	I317, A406, I403	
Q694		
Y695		E325
Q696		M324 backbone carbonyl
E697		R409

*Residues observed in the electron density map for PCF11 protomer A are listed.

Supplementary References

1. Laskowski, R.A. & Swindells, M.B. LigPlot+: multiple ligand-protein interaction diagrams for drug discovery. *J Chem Inf Model* **51**, 2778-86 (2011).
