# Supplementary material for: Structural basis for substrate recognition and chemical inhibition of oncogenic MAGE ubiquitin ligases
Source: Nat Commun. 2020 Oct 1;11:4931. doi: 10.1038/s41467-020-18708-x (PMC7529893; doi:10.1038/s41467-020-18708-x)
Supplement: Supplementary file 11 — Reporting Summary [file 41467_2020_18708_MOESM11_ESM.pdf]

nature research

Corresponding author(s): Patrick Ryan Potts Ph.D.

Last updated by author(s): Aug 15, 2020

Reporting Summary

Nature Research wishes to improve the reproducibility of the work that we publish. This form provides structure for consistency and transparency in reporting. For further information on Nature Research policies, see Authors & Referees and the Editorial Policy Checklist.

Statistics

For all statistical analyses, confirm that the following items are present in the figure legend, table legend, main text, or Methods section.

n/a	Confirmed	
☐	☒	The exact sample size (*n*) for each experimental group/condition, given as a discrete number and unit of measurement
☐	☒	A statement on whether measurements were taken from distinct samples or whether the same sample was measured repeatedly
☐	☒	The statistical test(s) used AND whether they are one- or two-sided<br>*Only common tests should be described solely by name; describe more complex techniques in the Methods section.*
☒	☐	A description of all covariates tested
☒	☐	A description of any assumptions or corrections, such as tests of normality and adjustment for multiple comparisons
☐	☒	A full description of the statistical parameters including central tendency (e.g. means) or other basic estimates (e.g. regression coefficient) AND variation (e.g. standard deviation) or associated estimates of uncertainty (e.g. confidence intervals)
☐	☒	For null hypothesis testing, the test statistic (e.g. *F*, *t*, *r*) with confidence intervals, effect sizes, degrees of freedom and *P* value noted<br>*Give P values as exact values whenever suitable.*
☒	☐	For Bayesian analysis, information on the choice of priors and Markov chain Monte Carlo settings
☒	☐	For hierarchical and complex designs, identification of the appropriate level for tests and full reporting of outcomes
☒	☐	Estimates of effect sizes (e.g. Cohen's *d*, Pearson's *r*), indicating how they were calculated

Our web collection on statistics for biologists contains articles on many of the points above.

Software and code

Policy information about availability of computer code

Data collection	No software was used for data collection
Data analysis	For RNAseq analysis: Low quality reads were filtered out using Trim Galore (v.0.6.5) and then aligned to the human genome (hg19/GRCh37) using STAR version 2.5.2b36. The mapped BAM files were converted into bedgraph format using bedtools version 2.17.037. To determine significant alternative polyadenylation events from RNA-seq data, we utilized previously described DaPars (v.0.9.1) (PMID: 25409906).<br><br>X-ray diffraction data were integrated and scaled using HKL2000 v716 (PMID: 27754618). The structure was solved by molecular replacement with Phaser v2.8 (PMID: 19461840). Iterative rounds of model building and refinement were performed with COOT v0.8.9 (PMID: 20383002) and Refmac v5.8.0257 (PMID: 15299926).<br><br>Graphpad Prism v7 was used for statistical analysis.

For manuscripts utilizing custom algorithms or software that are central to the research but not yet described in published literature, software must be made available to editors/reviewers. We strongly encourage code deposition in a community repository (e.g. GitHub). See the Nature Research guidelines for submitting code & software for further information.

Data

Policy information about availability of data

All manuscripts must include a data availability statement. This statement should provide the following information, where applicable:

- Accession codes, unique identifiers, or web links for publicly available datasets
- A list of figures that have associated raw data
- A description of any restrictions on data availability

The datasets generated during and/or analyzed during the current study are available from the corresponding author on reasonable request. Structural data that

October 2018

support the findings of this study have been deposited in PDB with the accession code PDB: 6WJH. Sequencing data that support the findings of this study have been deposited in the GEO with the accession code GSE148458. The source data underlying Figs. 1a, 1h, 2a, 2e,, 2f, 3a, 4c, 4e, 4g, 5d, 5h, 5i, and Supplemental Figs. 2a and 4e are provided as a Source Data file.

Field-specific reporting

Please select the one below that is the best fit for your research. If you are not sure, read the appropriate sections before making your selection.

☒ Life sciences ☐ Behavioural & social sciences ☐ Ecological, evolutionary & environmental sciences

For a reference copy of the document with all sections, see nature.com/documents/nr-reporting-summary-flat.pdf

Life sciences study design

All studies must disclose on these points even when the disclosure is negative.

Sample size	Sample sizes were determined using accepted methods in the field.<br><br>In vitro experiments were repeated at least 3 times with biological replicates. Sample sizes in each experiment were determined based on expected effect size and variability as well as the necessary amount of material suitable for a given assay.<br><br>Sample sizes for in vivo experiments were determined by power analysis. At least 6 mice were used per experimental group for xenograft assays.
Data exclusions	No data was excluded.
Replication	Experiments were independently replicated at least three time with similar outcomes.
Randomization	Samples and mice were randomly distributed into control and experimental groups.
Blinding	Does not apply to our study because we use all data for analysis.

Reporting for specific materials, systems and methods

We require information from authors about some types of materials, experimental systems and methods used in many studies. Here, indicate whether each material, system or method listed is relevant to your study. If you are not sure if a list item applies to your research, read the appropriate section before selecting a response.

Materials & experimental systems

n/a	Involved in the study	
☐	☒	Antibodies
☐	☒	Eukaryotic cell lines
☒	☐	Palaeontology
☐	☒	Animals and other organisms
☒	☐	Human research participants
☒	☐	Clinical data

Methods

n/a	Involved in the study	
☒	☐	ChIP-seq
☒	☐	Flow cytometry
☒	☐	MRI-based neuroimaging

Antibodies

Antibodies used	anti-Myc (Sigma, #C3956), anti-FLAG (Sigma, #F3165), anti-HUWE1 (Novus Biologicals, #NB 100-652), anti-PCF11 (Bethyl Laboratories, #A303-706A), anti-GAPDH (Cell Signaling Technology, #2118), anti-TRIM28 (Abcam, #ab22553), anti-AMPKα1 (Cell Signaling Technology, #2795), anti-MMS19 (Proteintech, #16015-1-AP) and Tb-anti-GST (Invitrogen, #PV3551). Secondary antibodies were: Donkey Anti-Rabbit IgG (GE Healthcare, NA934V) and Sheep Anti-Mouse IgG (GE Healthcare, NA931V). FLAG and TRIM28 primary antibodies were used at a dilution of 1:10,000 and Tb-anti-GST antibody was used at a dilution 1:720 and other primary antibodies were used at a dilution of 1:1000. All GE Healthcare secondary antibodies were used at a dilution of 1:5000.
Validation	All antibodies were validated by commercial sources for use in western blotting of human cell lines: anti-Myc (Sigma, #C3956); https://www.sigmaaldrich.com/content/dam/sigma-aldrich/docs/Sigma/Datasheet/3/c3956dat.pdf, anti-FLAG (Sigma, #F3165); https://www.sigmaaldrich.com/content/dam/sigma-aldrich/docs/Sigma/Datasheet/f3165dat.pdf, anti-HUWE1 (Novus Biologicals, #NB 100-652); https://www.novusbio.com/products/huwe1-antibody_nb100-652, anti-PCF11 (Bethyl Laboratories, #A303-706A); https://www.bethyl.com/product/A303-706A/PCF11+Antibody, anti-GAPDH (Cell Signaling Technology, #2118); https://www.cellsignal.com/products/primary-antibodies/gapdh-14c10-rabbit-mab/2118, anti-TRIM28 (Abcam, #ab22553); https://www.abcam.com/kap1-antibody-20c1-ab22553.html, anti-AMPKα1 (Cell Signaling Technology, #2795); https://www.cellsignal.com/products/primary-antibodies/ampka1-antibody/2795 and anti-MMS19 (Proteintech, #16015-1-AP); https://

www.ptglab.com/products/MMS19-Antibody-16015-1-AP.htm, Tb-anti-GST Antibody was used in a TR-FRET assay; https://www.thermofisher.com/document-connect/document-connect.html?url=https%3A%2F%2Fassets.thermofisher.com%2FTFS-Assets%2FLSG%2Fcertificate%2FCertificates-of-Analysis%2FCOA_PV3551_2068723F.pdf&title=MjA2ODcyM0Y=.

Eukaryotic cell lines

Policy information about cell lines

Cell line source(s)	HEK293FT (Thermo Fisher Scientific, Cat# R70007), HeLa (Clontech Laboratories, 631156) and DAOY (ATCC, Cat# HTB-186)
Authentication	STR analysis
Mycoplasma contamination	Mycoplasma contamination was routinely tested and confirmed negative before experimentation.
Commonly misidentified lines (See ICLAC register)	No commonly misidentified cell lines were used in the study

Animals and other organisms

Policy information about studies involving animals; ARRIVE guidelines recommended for reporting animal research

Laboratory animals	5 week old female NOD.Cg-Prkdcscid Il2rgtm1Wjl/SzJ: NOD scid gamma mice (The Jackson Laboratory, 005557; RRID: IMSR_JAX:005557)
Wild animals	No wild animals were used in the study
Field-collected samples	No field collected samples were used in the study
Ethics oversight	All studies were approved by the St. Jude Children's Research Hospital institutional review committee on animal safety.

Note that full information on the approval of the study protocol must also be provided in the manuscript.

October 2018
